# Association of the monocyte-to-lymphocyte ratio with HIV viral load and anemia in HIV-positive patients: a retrospective cross-sectional study

**DOI:** 10.3389/fcimb.2026.1916863

**Published:** 2026-09-28

**Authors:** Abdulaziz M. Almuqrin, Hamood AlSudais, Turky AlSulaiman, Maram S. Alharbi, Adel Alragas, Mousa H. Muqri, Hamad M. Almatrudi, Fatimah S. Alshahrani, Yazeed Alshuweishi

**Affiliations:** 1 Department of Clinical Laboratory Sciences, College of Applied Medical Sciences, King Saud University, Riyadh, Saudi Arabia; 2 King Saud Medical City, King Saud University, Riyadh, Saudi Arabia; 3 Department of Clinical Pharmacy, College of Pharmacy, King Saud University, Riyadh, Saudi Arabia; 4 Department of Internal Medicine, Infectious Diseases Unit, College of Medicine, King Saud University, Riyadh, Saudi Arabia; 5 Division of Infectious Diseases, Department of Internal Medicine, King Saud University, Riyadh, Saudi Arabia; 6 Infection Prevention and Control Department, King Saud University Medical City, King Saud University, Riyadh, Saudi Arabia

**Keywords:** anemia, HIV, inflammatory markers, monocyte-to-lymphocyte ratio, viral load

## Abstract

**Background:**

HIV is a chronic viral infection characterized by persistent immune activation and various clinical complications, including anemia, which has been associated with accelerated progression to AIDS. The monocyte-to-lymphocyte ratio (MLR) is an emerging complete blood count (CBC)-derived inflammatory biomarker, but its relevance to HIV viral burden and HIV-associated anemia remains insufficiently explored. This study evaluated the associations of MLR with HIV viral load and anemia among HIV-positive patients in Saudi Arabia.

**Materials and methods:**

Demographics, clinical, and laboratory data were retrospectively retrieved from 103 HIV-positive patients at King Khalid University Hospital between 2017 and 2025. Patients with missing clinical and key laboratory data required for the primary analyses, including hemoglobin concentration and HIV viral load, were excluded, resulting in a final cohort of 84 patients. The associations of MLR with HIV viral load and anemia were comprehensively evaluated using group comparisons, correlation analysis, regression analysis, and receiver operating characteristic (ROC) curve analysis.

**Results:**

MLR levels were significantly higher in patients with high HIV viral load (0.5: 0.267–0.8) than in those with low viral load (0.25:0.183–0.446). Similarly, anemic HIV-positive patients showed significantly elevated MLR levels (0.5: 0.259–0.848) compared with non-anemic patients (0.24:0.173–0.3905). Furthermore, among anemic HIV-positive patients, MLR was significantly higher in those with high viral load compared to their counterparts. MLR demonstrated modest discriminatory performance for high HIV viral load and anemia in the study cohort. In addition, elevated MLR was significantly associated with increased odds of high HIV viral load (OR = 3.72, p = 0.0052) and anemia (OR = 4.21, p = 0.0027).

**Discussion:**

MLR was significantly associated with high HIV viral load and anemia in HIV-positive individuals, suggesting its potential relevance as a simple and routinely available CBC-derived marker in this population. However, larger prospective multicenter studies are needed to further evaluate its potential clinical utility.

## 1 Introduction

Since it was discovered in 1983, Human immunodeficiency virus (HIV), the causative agent of acquired immunodeficiency syndrome (AIDS), has become one of the global health challenges, responsible for more than 40 million deaths worldwide (Pitchumoni and Brun, 2025). Although the Middle East and North Africa (MENA) region has the lowest prevalence of people living with HIV (PLWHIV), with an estimated 210,000 cases, recent reports indicate a sharp rise in new infections. Between 2010 and 2022, the region experienced a 116% increase in new HIV cases, the highest growth rate reported globally (Unaids, 2024). HIV causes progressive immune dysregulation, characterized by persistent immune activation and various systemic complications (Teer et al., 2025; Dumitrescu et al., 2026).

Hematological abnormalities are among the many clinical complications associated with HIV infection, with anemia being one of the most frequently observed (Damtie et al., 2021; Cao et al., 2022). Anemia is a major global health issue affecting billions of people across all age groups (Gallagher, 2022; Wacka et al., 2024). It is increasingly recognized as one of the leading causes of morbidity and mortality, particularly in patients with chronic and acute microbial infections (Isanaka et al., 2012; Yang et al., 2018; Abuga et al., 2020; Veronese et al., 2023). In chronic conditions, anemia is primarily driven by the persistent production of proinflammatory cytokines, which ultimately interfere with red blood cell (RBC) formation and life span, and restrict hemoglobin production (Begum and Latunde-Dada, 2019; Weiss et al., 2019). Numerous studies have linked anemia with the increased risk of progression of HIV to AIDS and death. This association may be partly attributed to chronic HIV-induced inflammation, which disrupts iron regulation and impairs red blood cell production (De Santis et al., 2011; Kamvuma et al., 2024).

Recently, new-generation inflammatory markers that are derived from complete blood count (CBC) have gained attention as useful markers for monitoring systemic inflammation in chronic infectious and inflammatory conditions (He et al., 2016; Koval et al., 2022; Dugăeşescu et al., 2025; Fang et al., 2025; Han et al., 2025; Man et al., 2025). Among these, the monocyte-to-lymphocyte ratio (MLR) has been highlighted in various studies for its potential to assess disease severity and predict outcomes. Qiu et al. reported that elevated MLR was significantly associated with an increased risk of all-cause mortality among ICU patients with type 2 diabetes and chronic kidney disease (Qiu et al., 2023). Another study, involving 399 stroke-associated pneumonia patients (SAP), found that MLR was significantly correlated with SAP and the prediction of severe pneumonia in this population (Cao et al., 2021). Further studies have linked elevated MLR with a higher risk of cardiovascular complications in patients with chronic kidney disease and with increased COVID-19-related mortality (Dávila-Collado et al., 2021; Oh et al., 2022).

Despite growing interest in CBC-derived inflammatory indices, evidence regarding the clinical relevance of MLR in HIV-positive patients remains limited, particularly in relation to both anemia and virologic burden. This gap is clinically important because anemia is a common complication of HIV infection that is associated with adverse outcomes, while viral load reflects ongoing disease activity.

This study aims to evaluate the associations of MLR with anemia and HIV viral load in HIV-positive patients. To the best of our knowledge, this is the first report from Saudi Arabia and among the first from the MENA region to address these relationships.

## 2 Methods

### 2.1 Data collection and study design

Following approval from the Institutional Review Board (IRB) at King Khalid University Hospital (KKUH), Riyadh, Saudi Arabia (IRB No. E-24-9143), the demographics, clinical, and laboratory records of 103 HIV-positive patients were retrospectively retrieved from the databases of the Infectious Diseases and General Internal Medicine Clinics for the period 2017–2025. Nineteen subjects were excluded, resulting in a final HIV-positive study cohort of 84 individuals, comprising 68 males and 16 females, as illustrated in **Figure 1**. The excluded records lacked sufficient key laboratory data, including missing Hb and HIV viral load measures, which were required to define anemia status and viral load category. Males with Hb levels of ≤12.9 g/dL and females with levels of ≤11.4 g/dL were classified as anemic individuals (Bakr et al., 2022). High HIV viral load (H-VL) was defined as ≥100,000 copies/mL, a threshold commonly used to indicate high-level viremia in HIV laboratory monitoring (Vajpayee and Mohan, 2011). Documented treatment status was retrieved from the electronic medical records as a binary variable indicating whether each patient had documented HIV treatment at the time of data extraction. However, detailed ART-related information, including regimen type, treatment duration, adherence, and treatment history, was not consistently available.

**Figure 1:**
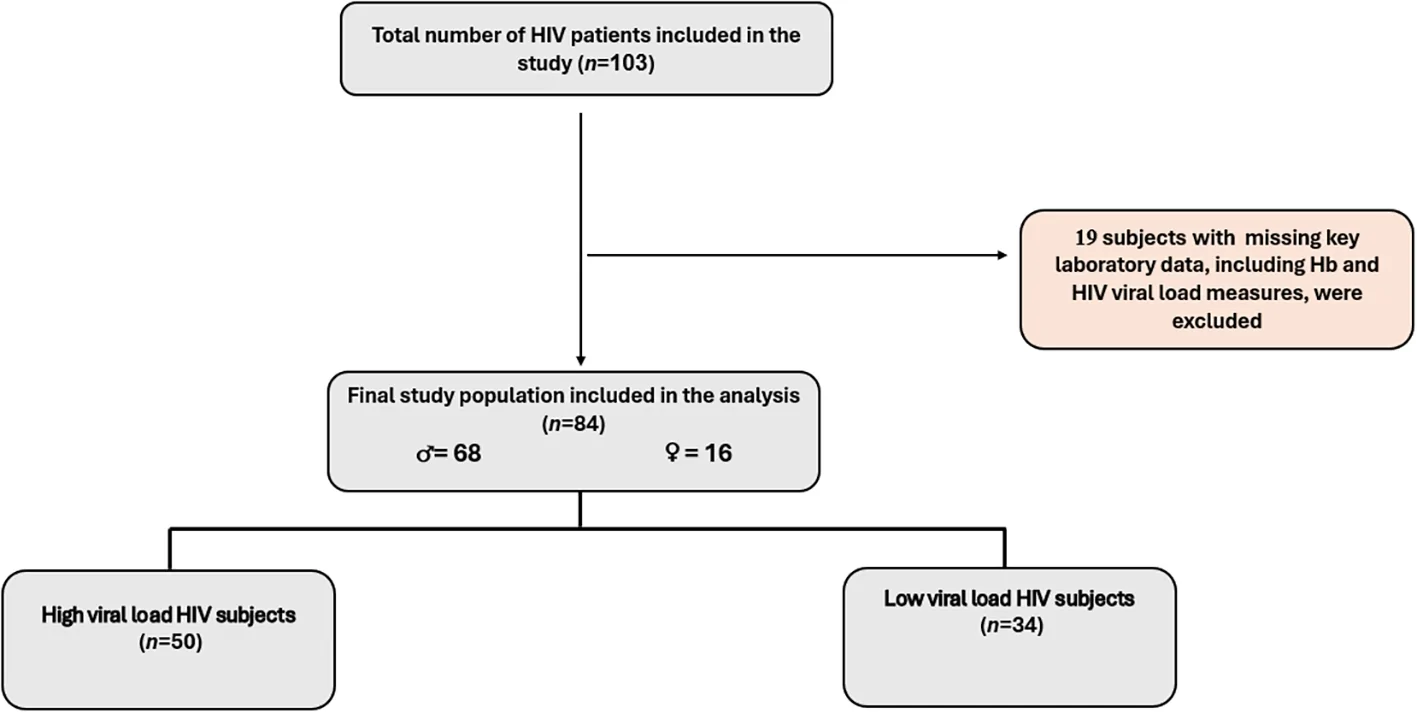
Flow chart of the study design.

Based on receiver operating characteristic (ROC) curve analysis, the optimal MLR cut-off value, achieving the highest sensitivity and specificity, was determined to be >0.354. Therefore, MLR values above 0.354 were considered high. MLR was calculated as the ratio of the absolute monocyte count to the absolute lymphocyte count.

### 2.2 Statistical analysis

Since the data weren’t normally distributed, nonparametric tests were used for statistical analysis. Comparisons between two groups were performed using the Mann–Whitney U test. Continuous variables were presented as medians with interquartile ranges (IQRs), whereas categorical data were summarized as percentages.

The discriminatory performance of MLR in distinguishing anemic from non-anemic subjects and patients with high from those with low HIV viral load was evaluated using ROC curve analysis, with the area under the curve (AUC) used to quantify discriminatory performance. Risk assessment analyses, including prevalence ratios (PRs) and odds ratios (ORs), were performed using MedCalc Statistical Software version 20.218 (MedCalc Software Ltd., Ostend, Belgium). Spearman’s correlation was used to assess the relationships between the MLR and Hb concentrations and HIV viral load in the study cohort. For continuous analyses, HIV viral-load values were expressed as log10 HIV RNA copies/mL. Linear regression analyses were performed to evaluate the associations of MLR with HIV viral-load category and anemia status. Model 1 was unadjusted, whereas Model 2 was adjusted for prespecified covariates, including age and sex. Given the relatively small sample size, covariates were selected *a priori* based on clinical relevance, and laboratory markers and medication-use variables were not included in the adjusted model to reduce the risk of overfitting. Stepwise variable selection was not used. Results were reported as β coefficients with 95% confidence intervals. Descriptive statistics, Mann–Whitney U tests, Spearman correlation analyses, ROC curve analyses, and regression analyses were performed using GraphPad Prism version 10.0.01 (GraphPad Software, Inc., San Diego, CA, USA). P-values < 0.05 were considered statistically significant.

## 3 Results

### 3.1 Baseline characteristics of the study subjects

The present study included 84 HIV-positive patients, comprising 68 males (81.0%) and 16 females (19.0%). As shown in **Table 1**, the study population was stratified according to HIV viral load into the High-VL group (n = 50), which included 42 males (84.0%) and 8 females (16.0%), and the Low-VL group (n = 34), which included 26 males (76.47%) and 8 females (23.53%). Males therefore constituted the majority of participants in both groups. Age, documented medication, and laboratory parameters are summarized in **Table 1**. No significant differences in age or laboratory parameters were observed between the High-VL and Low-VL groups.

**Table 1:** Baseline characteristics of the study subjects.

Variables	High viral load	Low viral load	P-value
Demographics			
Age (years)	35 (29.75-49)	32 (25-39.25)	0.0515
Gender (males), n (%)	42 (84%)	26 (76.47%)	-
Gender (females), n (%)	8 (16%)	8 (23.53%)	-
Lab parameters			
Hb (g/dL)	10.90 (9.625-13.25)	12.55 (10.30-14.00)	0.1337
Chloride (mmol/L)	103 (100.6-107)	104 (100-105)	0.8746
BUN (mmol/L)	4.00 (3.00-5.45)	4.20 (2.975-5.250)	0.9908
ALT (U/L)	29.8 (18.15-58.85)	31.50 (19.40-42.25)	0.9541
Creatinine (umol/L)	63 (51-80.50)	75.50 (59-84.25)	0.1552
Medications			
acyclovir, n (%)	8 (16%)	6 (17.6%)	-
daclatasvir	0 (0%)	1 (3%)	-
efavirenz	0 (0%)	1 (3%)	-
ganciclovir	14 (28%)	3 (8.8%)	-
zidovudine	0 (0%)	3 (8.8%)	-
lamivudine-zidovudine	0 (0%)	1 (3%)	-
sofosbuvir	0 (0%)	1 (3%)	-
valACYclovir	8 (16%)	2 (6%)	-
valGANciclovir	23 (46%)	4 (11.7%)	-

### 3.2 Elevated MLR levels in patients with high HIV viral load

As shown in **Figure 2**, the disparity in MLR levels between the Low-VL and High-VL groups was evaluated. MLR levels were significantly higher in the High-VL group (0.5: 0.267-0.8) compared with the Low-VL group in the overall study population (0.25: 0.1835-0.446) (**Figure 2A**). Sex-stratified analyses showed that the increase in MLR was statistically significant among male patients, whereas the corresponding difference did not reach statistical significance among female patients (**Figures 2B, C**). However, because of the small number of female participants, these sex-stratified findings should be considered exploratory.

**Figure 2:**
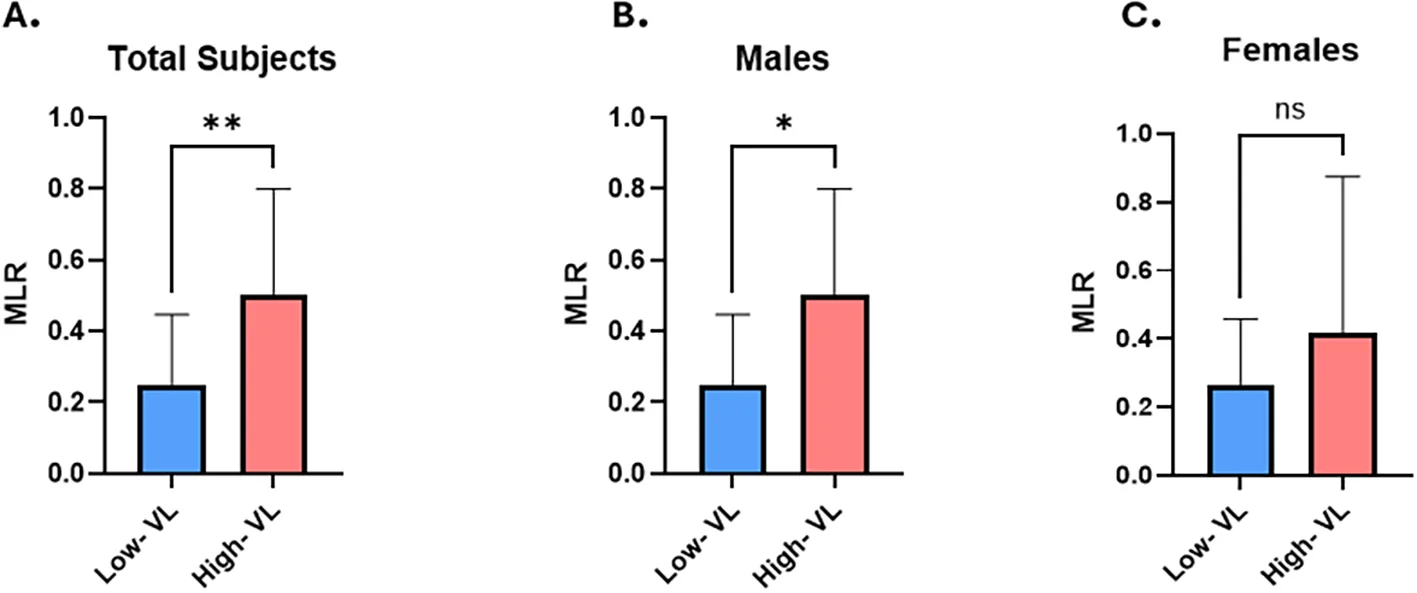
Comparison of MLR measures according to HIV viral load in the study population. Differences in MLR levels between the low HIV viral load and high HIV viral load groups are shown for total study subjects in **(A)**, for male subjects in **(B)**, and for females in **(C)**.

### 3.3 HIV viral load is higher in patients with elevated MLR

The association between MLR and HIV viral load in the study cohort was further assessed by comparing log10 HIV RNA copies/mL values across MLR categories. As shown in **Figure 3**, HIV viral load levels were significantly higher in subjects with high MLR (H-MLR) levels (5.597: 4.99-6.236) compared with those with normal MLR (N-MLR) measures (4.878: 3.455-5.647) in the overall population (**Figure 3A**). In exploratory sex-stratified analyses, this difference remained statistically significant among male patients (**Figure 3B**), whereas it did not reach statistical significance among female patients (**Figure 3C**).

**Figure 3:**
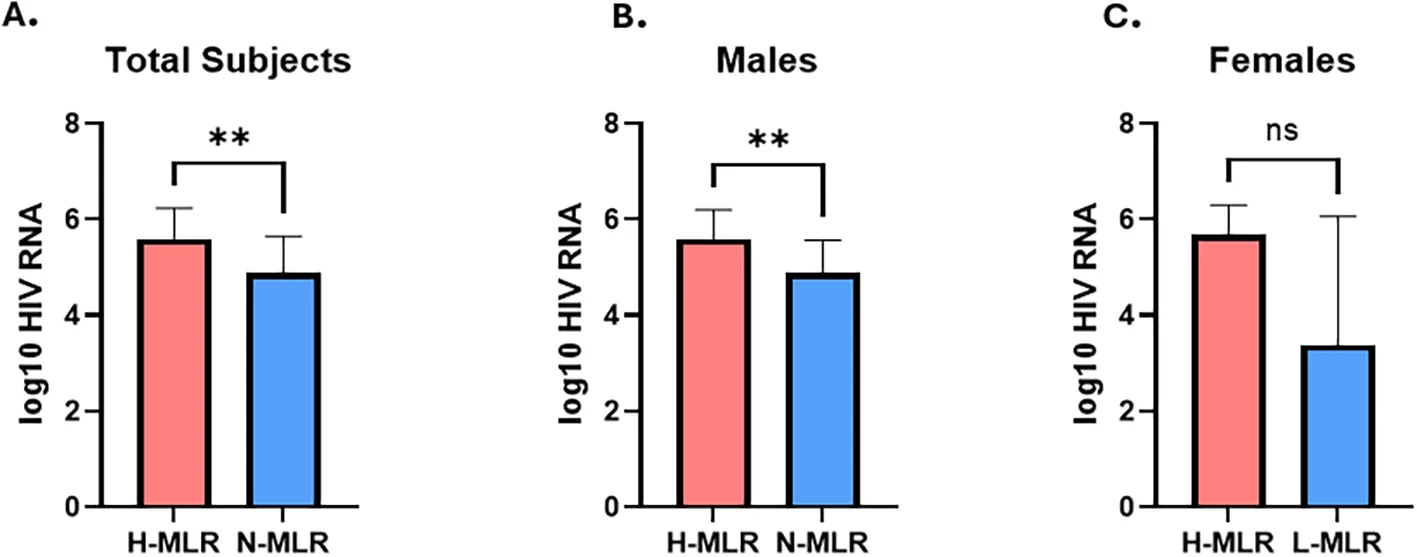
The variations in HIV load levels in light of MLR measures in the HIV-positive patients. The difference in log10 HIV RNA levels in light of MLR in total study subjects is shown in **(A)**, in males **(B)**, and in female participants **(C)**.

### 3.4 MLR measures are elevated in anemic HIV patients

To investigate the relationship between MLR and anemia in HIV patients, differences in MLR levels between anemic and non-anemic individuals were evaluated. MLR levels were significantly higher in the anemic group (0.5: 0.2585-0.8485) compared with the non-anemic group (0.24: 0.173-0.3905) in the overall study population (**Figure 4A**). In exploratory sex-stratified analyses, this difference remained statistically significant when males were analyzed separately (0.5: 0.2703-0.966 vs. 0.268: 0.1955-0.4218) (**Figure 4B**) but not in females (**Figure 4C**).

**Figure 4:**
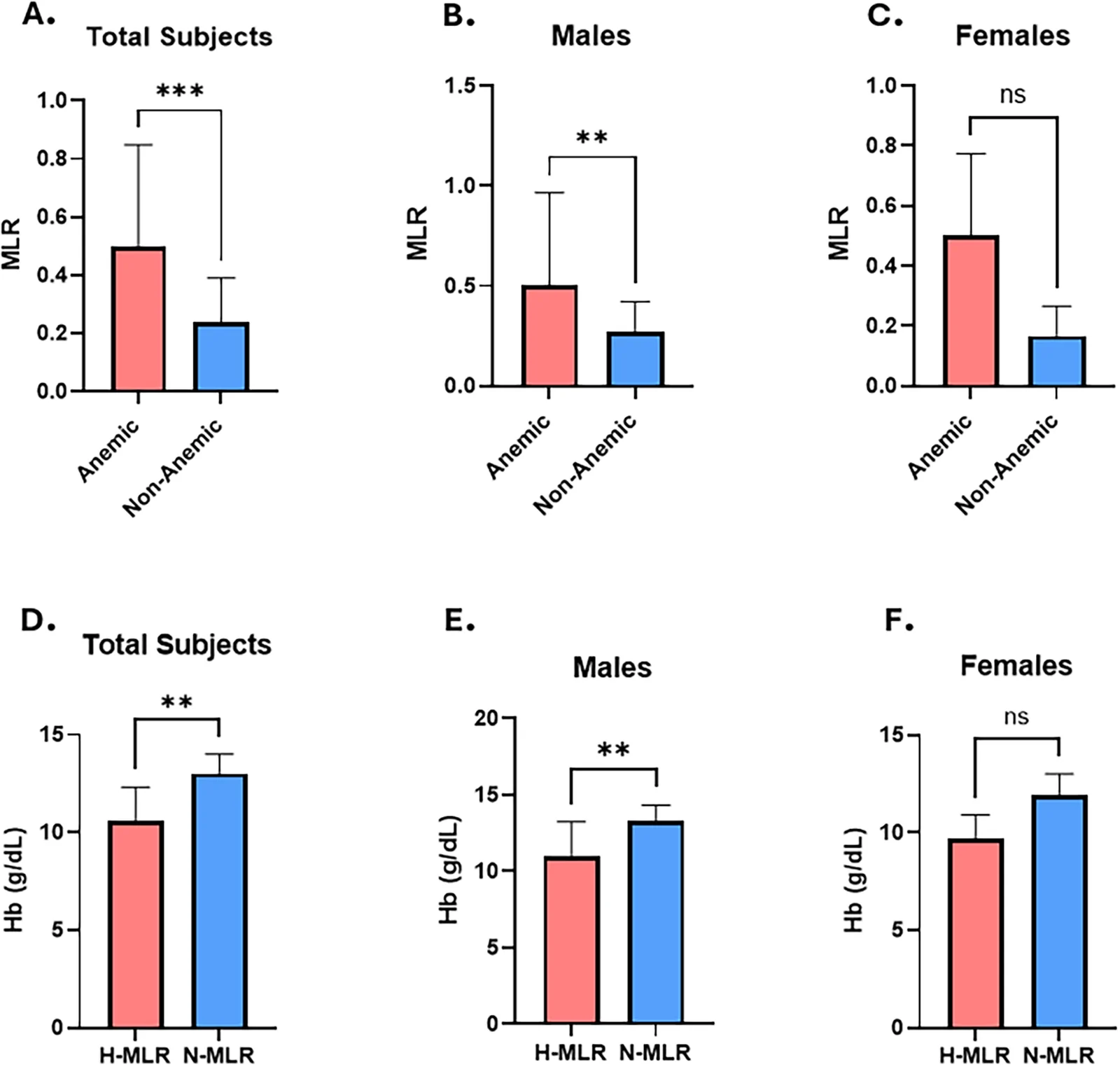
Evaluation of MLR measures in relation to Hb concentrations in HIV positive patients. Comparisons of MLR levels between anemic and non-anemic subjects are shown for the overall population **(A)**, male participants **(B)**, and females **(C)**. The change in Hb concentrations in light of MLR levels is presented for both genders **(D)**, males **(E)**, and females **(F)**.

Additionally, Hb concentrations were compared according to MLR categories. Hb levels were significantly lower in subjects with high MLR (10.6: 9.1-12.3) compared with those with normal MLR levels (13: 10.75-14) in the overall cohort (**Figure 4D**). This pattern persisted in male patients (11: 9.325-13.2 vs. 13.25: 11.03-14.3) (**Figure 4E**), whereas no significant difference in Hb levels was observed among female subjects in the sex-stratified exploratory analyses (**Figure 4F**).

### 3.5 Association of MLR with concurrent anemia and high HIV viral load

To further explore the relationship between anemia, HIV viral load, and MLR, subgroup analyses were performed according to anemia status and viral load category. Among patients with high HIV viral load, MLR levels were significantly higher in anemic subjects (0.6335: 0.333-1.00) compared with non-anemic subjects (0.293: 0.173-0.4365) (**Figure 5A**).

**Figure 5:**
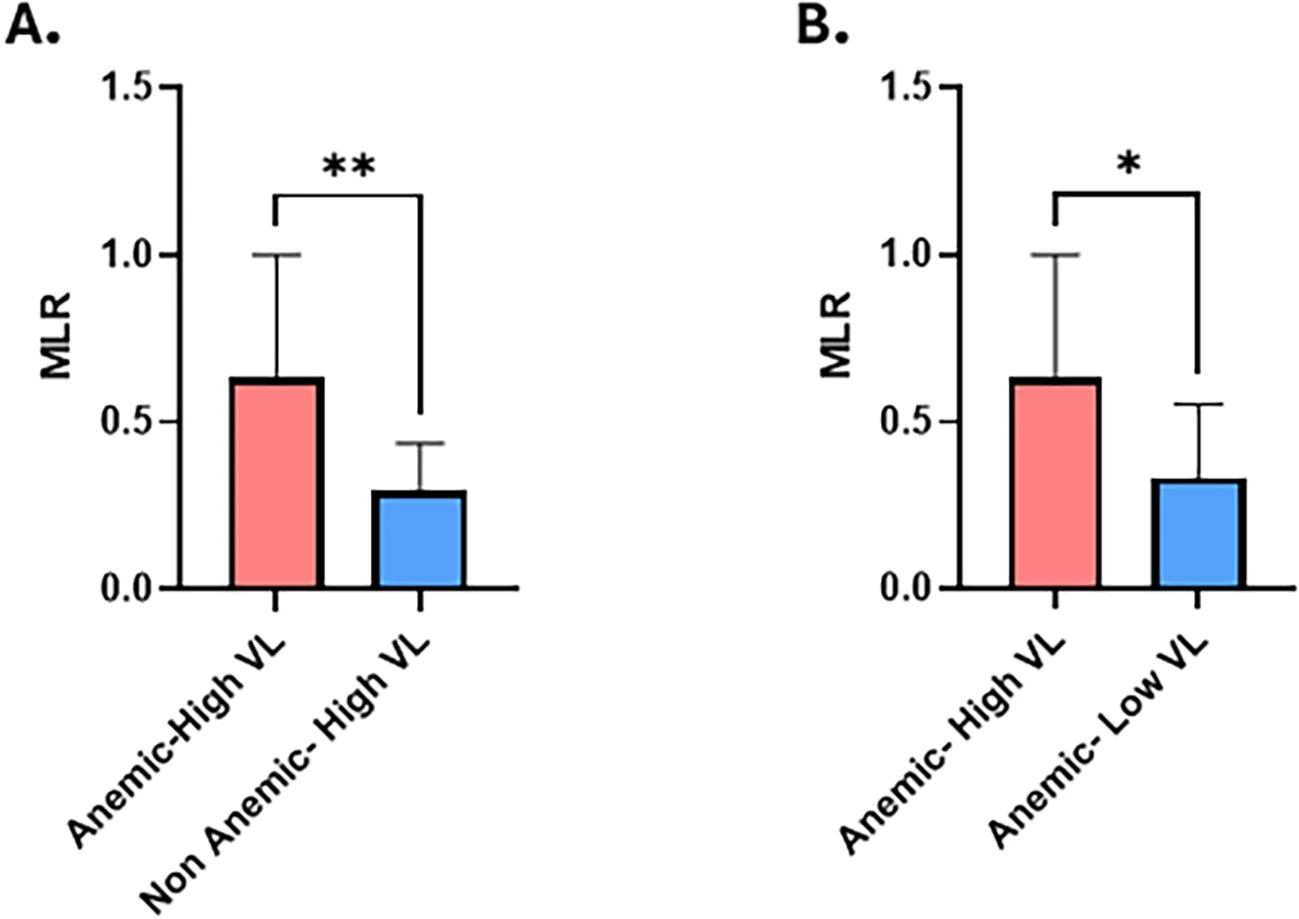
Combined effects of anemia and HIV viral load on MLR in the study patients. Comparisons of MLR levels between anemic and non-anemic subjects within the high HIV viral load group are shown in **(A)**. Differences in MLR measures between anemic subjects with high HIV viral load and anemic subjects with low viral load are shown in **(B)**. * p < 0.05; ** p < 0.01.

When the analysis was restricted to anemic patients, those with high viral load exhibited significantly higher MLR levels than those with low viral load (**Figure 5B**), showing that higher MLR levels were associated with the coexistence of anemia and high HIV viral load.

### 3.6 Distribution of elevated MLR by HIV viral load and anemia status

As detailed in **Table 2**, the prevalence of elevated MLR measures was evaluated across HIV viral load and anemia subgroups. Elevated MLR was more prevalent among patients with H-VL compared with those with Low-VL (64% vs. 32.35%). When stratified by anemia, elevated MLR levels were more frequently observed in anemic subjects than in non-anemic subjects (64.71% vs. 30.30%).

**Table 2:** Prevalence of elevated MLR measures across HIV viral load and anemia subgroups.

Parameter	Prevalence (%)	
	H-MLR	N-MLR
H-viral load	64%	36%
N-viral load	32.35%	67.65%
Anemic	64.71%	35.29%
Non-Anemic	30.30%	69.70%
Anemic and H-viral load	75.76%	24.24%
Anemic and N-viral load	44.40%	55.56%

Notably, the highest prevalence of elevated MLR was observed among anemic patients with H-VL (75.76%), compared with anemic patients with Low-VL (44.40%), highlighting a combined effect of anemia and HIV viral load on MLR measures in the study cohort.

### 3.7 Correlation of MLR with HIV viral load and Hb concentrations in the study cohort

The correlation analysis demonstrated a weak but statistically significant association between MLR and HIV viral load in the overall study population (r = 0.337, 95% CI: 0.1259 to 0.5190, p = 0.0017) (**Table 3**).

**Table 3:** Correlation of MLR with HIV load and Hb concentrations.

Marker	Variables	r	95% CI	P-value
*MLR*	Both genders			
	HIV viral load (log10 HIV RNA copies/mL)	0.337	0.1259 to 0.5190	*0.0017*
	Hb (g/dL)	-0.1955	-0.3988 to 0.02618	*0.074*
	*Males*			
	HIV viral load (log10 HIV RNA copies/mL)	0.31	0.06951 to 0.5155	0.0102
	Hb (g/dL)	-0.4339	-0.6138 to -0.2112	0.0002
	*Females*			
	HIV viral load (log10 HIV RNA copies/mL)	0.4458	-0.08010 to 0.7775	0.084
	Hb (g/dL)	-0.6327	-0.8631 to -0.1841	0.01

MLR remained significantly positively correlated with HIV viral load in male patients (r = 0.31, p = 0.0102). In addition, a moderate inverse correlation was observed between MLR and Hb concentration in males (r = -0.4339, p = 0.0002). Among female patients, MLR showed a significant inverse correlation with Hb concentration (r = -0.6327, 95% CI: −0.8631 to −0.1841, p = 0.01), whereas its correlation with HIV viral load did not reach statistical significance.

### 3.8 Risk association of elevated MLR with high HIV viral load and anemia in HIV-positive patients

As revealed in **Table 4**, risk assessment analysis demonstrated that elevated MLR was significantly associated with a higher prevalence of high HIV viral load (PR = 1.97, 95% CI = 1.16–3.35, *p* = 0.0114) and anemia (PR = 2.13, 95% CI = 1.22–3.72, *p* = 0.0075) in the study cohort. In addition, subjects with elevated MLR had significantly higher odds of presenting with high HIV viral load (OR = 3.717, 95% CI = 1.47–9.34, *p* = 0.0052) and anemia (OR = 4.21, 95% CI = 1.64–10.77, *p* = 0.0027) compared with those with normal MLR levels.

**Table 4:** Risk assessment of elevated MLR in relation to high HIV viral load and anemia.

Marker	Condition	Measure	Score	95% CI	z statistic	p-value
MLR	H-HIV load	*PR*	1.97	1.1659–3.3562	2.529	0.0114
		*OR*	3.7172	1.4787–9.3444	2.792	0.0052
	Anemia	*PR*	2.13	1.2249–3.7223	2.676	0.0075
		*OR*	4.2167	1.6494–10.7798	3.005	0.0027

### 3.9 Discriminatory performance of MLR for high HIV viral load and anemia in the study cohort

The discriminatory performance of MLR for distinguishing patients with high HIV viral load from those with low viral load and anemic from non-anemic participants was assessed. MLR showed a modest discriminatory performance for high HIV viral load (AUC = 0.68, 95% CI: 0.56 to 0.79, P = 0.005) (**Figure 6A**) and anemia (AUC = 0.74, 95% CI: 0.63 to 0.84, P = 0.002) (**Figure 6B**) in the study cohort.

**Figure 6:**
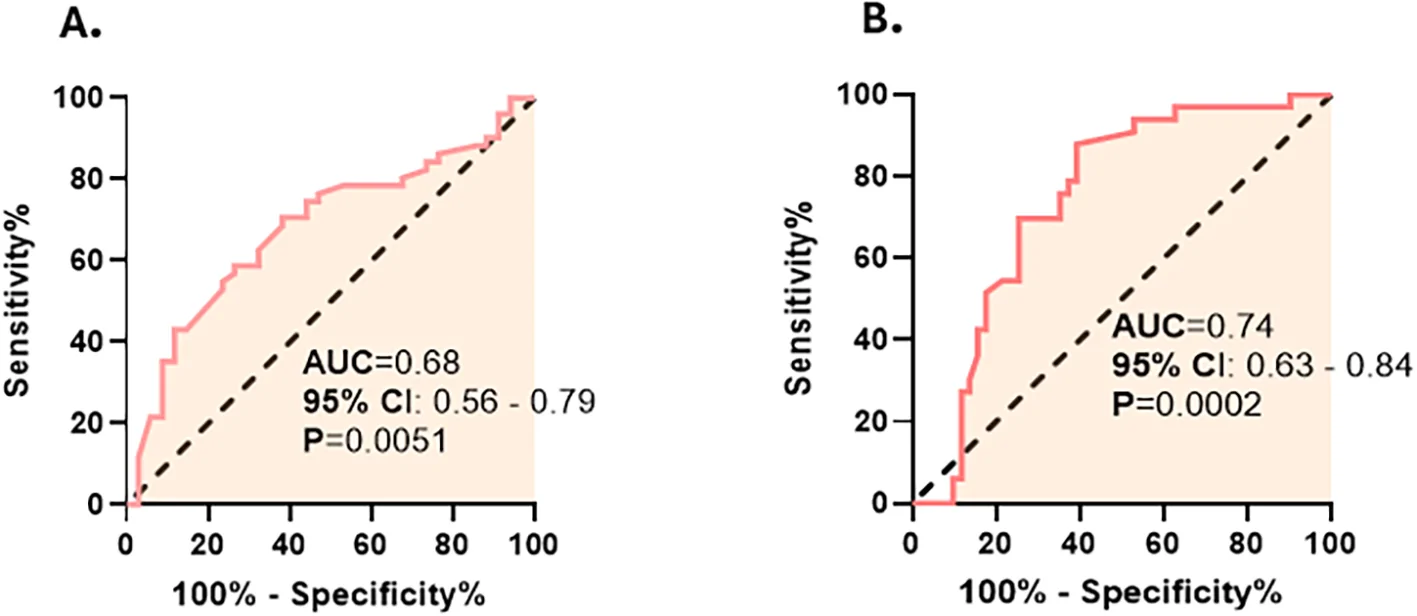
Discriminatory performance of MLR for high HIV viral load and anemia in the study cohort. ROC curves of MLR for distinguishing patients with high HIV viral load from those with low viral load **(A)** and anemic from non-anemic participants **(B)** are shown.

### 3.10 Association of MLR with HIV viral load and anemia status in regression analyses

The regression analysis showed that HIV viral load status was significantly associated with increased MLR in the unadjusted model (β = 0.2204, 95% CI: 0.02566 to 0.4151, p = 0.027) (**Table 5**). After adjustment for age and sex, this association was slightly attenuated (β = 0.1672, 95% CI: -0.03128 to 0.3658, p = 0.0975). Anemia status was significantly associated with MLR in the unadjusted model (β = - 0.3396, 95% CI: - 0.5269 to −0.1522, p = 0.0005), and this association remained statistically significant after adjustment for age and gender (β = -0.3183, 95% CI: -0.5044 to -0.1322, p = 0.001).

**Table 5:** Regression analysis of MLR with HIV viral-load category and anemia status.

Marker	Variable	Model	β	95% CI	p-value
MLR	Viral load	Model 1	0.2204	0.02566 to 0.4151	0.027
		Model 2	0.1672	-0.03128 to 0.3658	0.0975
	Hb	Model 1	-0.3396	-0.5269 to -0.1522	0.0005
		Model 2	-0.3183	-0.5044 to -0.1322	0.001

Model 1: unadjusted. Model 2: adjusted for age and sex.

## 4 Discussion

This study aimed to explore the associations of MLR with HIV viral load and anemia in a cohort of HIV-positive patients in Saudi Arabia. Given that viral load reflects ongoing HIV disease activity, and anemia is a frequent hematological complication associated with HIV progression and poor outcomes, MLR may provide a simple marker linking virologic burden with hematologic disturbance in HIV patients. The results of this study showed that MLR was significantly associated with high HIV viral load and anemia, and that MLR measures were highest among patients with concurrent anemia and high HIV viral activity. These findings suggest that MLR may serve as a simple marker reflecting both virologic burden and hematologic disturbance in HIV-positive individuals.

The increased MLR levels observed in patients with high HIV viral load may be explained by persistent HIV replication, which promotes chronic immune activation and alters monocyte dynamics during infection. It has been reported that HIV infection is associated with the expansion of circulating CD16+ monocyte subsets, which are more permissive to HIV infection and closely related to disease progression, while progressive infection is also characterized by lymphocyte depletion and immune dysregulation. Accordingly, elevated MLR may reflect the combined effect of enhanced monocyte-driven inflammation and relative lymphocyte disturbance in patients with greater virologic burden (Oramasionwu et al., 2012; Campbell et al., 2014; Mayito et al., 2020). Taken together, previous evidence supports the biological plausibility of the observed association between elevated MLR and higher HIV viral load in the present cohort. In addition, the present study showed a significant increase in MLR among anemic HIV-positive patients compared with their non-anemic counterparts. Anemia is a frequent complication of HIV infection and has been linked to disease progression and increased mortality risk (Gelaw et al., 2021; Jin et al., 2023). The higher MLR levels observed in anemic patients in the present study suggest that this marker may reflect the inflammatory and immunohematologic disturbances that accompany anemia in HIV infection.

Another notable finding of the present study was that MLR was highest in patients with concurrent anemia and high HIV viral load, suggesting that MLR may reflect the compounded burden arising from the coexistence of hematologic impairment and greater virologic burden. This interpretation is consistent with previous studies showing that anemia in HIV is associated with higher HIV RNA levels and more advanced disease, characterized by chronic inflammation and high viral replication (Mata-Marín et al., 2010; Tesfaye et al., 2023) The association between MLR and anemia observed in the present study is also supported by previous work by Gatechompol et al., who examined the impact of anemia severity on the risk of developing tuberculosis in HIV-positive patients following antiretroviral therapy. That study showed that both Hb and MLR were associated with an increased risk of tuberculosis, and that combining the two markers strengthened risk stratification for identifying high-risk individuals (Gatechompol et al., 2024). Another large longitudinal U.S. study of 32,867 HIV-positive patients linked anemia to more severe HIV disease and increased mortality risk (Sullivan et al., 1998). In agreement with these findings, our results suggest that elevated MLR may reflect the greater inflammatory and clinical burden associated with the coexistence of anemia and increased virologic burden in HIV infection. This interpretation is consistent with previous reports linking anemia in HIV to various HIV-related complications and mortality (Kerkhoff et al., 2015; Gelaw et al., 2021; Wang et al., 2021; Jin et al., 2023).

Several mechanisms may explain the observed relationship between anemia, high HIV viral load, and elevated MLR in the present study. HIV infection can contribute to anemia both directly and indirectly through chronic immune activation, inflammatory cytokine production, and disruption of hematopoiesis. Previous studies have suggested that HIV may impair hematopoietic progenitor and stem cell survival and differentiation, including erythroid maturation, thereby reducing red blood cell production (Zauli et al., 1992; Alexaki and Wigdahl, 2008; Carter et al., 2011; McNamara and Collins, 2011; Gibellini et al., 2013; Morini et al., 2016; Renelt et al., 2022; Kamvuma et al., 2024) In parallel, inflammatory mediators such as TNF-α and IL-6 have been linked to HIV-related anemia through suppression of erythropoiesis, altered iron metabolism, and increased hepcidin production, which restricts iron availability for red blood cell synthesis (Ellaurie and Rubinstein, 1995; Boulware et al., 2011; Akchurin et al., 2018; Ketut Agus Somia et al., 2019; Ketut Agus Somia et al., 2019).

Importantly, monocyte activation has also been implicated in HIV-related complications, including anemia, and markers of monocyte/macrophage activation, such as neopterin, have been shown to correlate with anemia severity in HIV-positive patients (Fuchs et al., 1993; Lipshultz et al., 2015). Zhang et al., demonstrated that the activation of monocytes in response to inflammatory cytokines triggers an increase in hepcidin levels, which leads to ferroportin degradation, restricting iron absorption and promoting iron sequestration, thereby contributing to chronic anemia in HIV-positive patients (Zhang and Rovin, 2010). Therefore, in patients with greater virologic burden, persistent immune activation may simultaneously enhance monocyte-driven inflammation and worsen anemia-related hematologic disturbances, providing a plausible explanation for the elevated MLR observed in patients with concurrent high HIV viral load and anemia. However, these mechanisms were not directly assessed in the present study and should be confirmed in future mechanistic studies.

Sex-stratified analyses in this report indicated that the relationships of MLR with HIV viral load and anemia were more apparent in male patients than in females. One possible explanation is that sex-based differences in immune regulation, potentially influenced by estrogens and androgens, may modulate monocyte-related inflammatory responses and contribute to higher MLR values in males (Buttle et al., 2021). However, these findings should be interpreted cautiously because the female subgroup was small and underpowered. Therefore, the observed sex-stratified patterns should be considered exploratory and require confirmation in larger cohorts with adequate representation of both sexes.

To our knowledge, this is the first study from Saudi Arabia and one of the first from the MENA region to evaluate the association of a CBC-derived inflammatory marker with both HIV viral load and anemia in HIV-positive patients. The present findings provide important preliminary evidence supporting the potential relevance of MLR as a simple marker of HIV-related virologic and hematologic burden. Nevertheless, this study has a few limitations. Its retrospective design limits causal interpretation, and the relatively small sample size, particularly in sex-stratified analyses, may have reduced statistical power. In addition, 19 of the initially identified 103 HIV-positive records were excluded because of insufficient key laboratory data, including missing Hb and HIV viral-load data. Because these records lacked sufficient baseline information, included and excluded patients could not be compared, and potential selection bias related to complete-case analysis cannot be excluded. Detailed ART-related information was unavailable, including ART status, regimen, duration, adherence, treatment interruption, treatment-naïve status, and virologic failure; therefore, residual confounding related to ART exposure and treatment response cannot be excluded. In addition, the lack of important clinical variables, such as CD4 count, limited further mechanistic interpretation and adjustment for potential confounders.

## Data Availability

The original contributions presented in the study are included in the article/supplementary material. Further inquiries can be directed to the corresponding author.

## References

[B1] Abuga K. M. Muriuki J. M. Williams T. N. Atkinson S. H. (2020). How severe anaemia might influence the risk of invasive bacterial infections in African children. Int. J. Mol. Sci. 21, 6976. doi: 10.3390/IJMS21186976 32972031 PMC7555399

[B2] Akchurin O. Patino E. Dalal V. Meza K. Bhatia D. Brovender S. . (2018). Interleukin-6 contributes to the development of anemia in juvenile CKD. Kidney Int. Rep. 4, 470. doi: 10.1016/J.EKIR.2018.12.006 30899874 PMC6409399

[B3] Alexaki A. Wigdahl B. (2008). HIV-1 infection of bone marrow hematopoietic progenitor cells and their role in trafficking and viral dissemination. PLoS Pathog. 4, e1000215. doi: 10.1371/JOURNAL.PPAT.1000215 19112504 PMC2603331

[B4] Bakr S. AlFattani A. Al-Nounou R. Bakshi N. Khogeer H. Alharbi M. . (2022). Hematologic reference intervals for healthy adult Saudis in Riyadh. Ann. Saudi Med. 42, 191. doi: 10.5144/0256-4947.2022.191 35658586 PMC9167458

[B5] Begum S. Latunde-Dada G. O. (2019). Anemia of inflammation with an emphasis on chronic kidney disease. Nutrients 11, 2424. doi: 10.3390/NU11102424 31614529 PMC6835368

[B6] Boulware D. R. Hullsiek K. H. Puronen C. E. Rupert A. Baker J. V. French M. A. . (2011). Higher levels of CRP, D-dimer, IL-6, and hyaluronic acid before initiation of antiretroviral therapy (ART) are associated with increased risk of AIDS or death. J. Infect. Dis. 203, 1637–1646. doi: 10.1093/INFDIS/JIR134 21592994 PMC3096784

[B7] Buttle T. S. Hummerstone C. Y. Billahalli T. Ward R. J. B. Barnes K. E. Marshall N. J. . (2021). The monocyte-to-lymphocyte ratio: Sex-specific differences in the tuberculosis disease spectrum, diagnostic indices and defining normal ranges. PLoS One 16. doi: 10.1371/JOURNAL.PONE.0247745 34460817 PMC8405018

[B8] Campbell J. H. Hearps A. C. Martin G. E. Williams K. C. Crowe S. M. (2014). The importance of monocytes and macrophages in HIV pathogenesis, treatment, and cure. AIDS 28, 2175. doi: 10.1097/QAD.0000000000000408 25144219 PMC6331181

[B9] Cao F. Wan Y. Lei C. Zhong L. M. Lei H. T. Sun H. . (2021). Monocyte-to-lymphocyte ratio as a predictor of stroke-associated pneumonia: A retrospective study-based investigation. Brain Behav. 11, e02141. doi: 10.1002/BRB3.2141 33942561 PMC8213641

[B10] Cao G. Wang Y. Wu Y. Jing W. Liu J. Liu M. (2022). Prevalence of anemia among people living with HIV: A systematic review and meta-analysis. EClinicalMedicine 44, 101283. doi: 10.1016/J.ECLINM.2022.101283 35128369 PMC8803600

[B11] Carter C. C. McNamara L. A. Onafuwa-Nuga A. Shackleton M. Riddell J. Bixby D. . (2011). HIV-1 utilizes the CXCR4 chemokine receptor to infect multipotent hematopoietic stem and progenitor cells. Cell Host Microbe 9, 223–234. doi: 10.1016/J.CHOM.2011.02.005/ATTACHMENT/8E8F5AF6-A2B5-443C-945F-B1E0FF306496/MMC1.PDF 21402361 PMC3102232

[B12] Damtie S. Workineh L. Kiros T. Eyayu T. Tiruneh T. (2021). Hematological abnormalities of adult HIV-infected patients before and after initiation of highly active antiretroviral treatment at Debre Tabor Comprehensive Specialized Hospital, Northcentral Ethiopia: A cross-sectional study. HIV AIDS (Auckl) 13, 477. doi: 10.2147/HIV.S308422 33976573 PMC8106447

[B13] Dávila-Collado R. Jarquín-Durán O. Solís-Vallejo A. Nguyen M. A. Espinoza J. L. (2021). Elevated monocyte to lymphocyte ratio and increased mortality among patients with chronic kidney disease hospitalized for COVID-19. J. Pers. Med. 11, 224. doi: 10.3390/JPM11030224 33809858 PMC8004261

[B14] De Santis G. C. Brunetta D. M. Vilar F. C. Brandão R. A. de Albernaz Muniz R. Z. de Lima G. M. N. . (2011). Hematological abnormalities in HIV-infected patients. Int. J. Infect. Dis. 15, e808–e811. doi: 10.1016/j.ijid.2011.08.001 21880530

[B15] Dugăeşescu M. Andrei-Bitere I. Baciu M. R. Dănescu E. Liţescu A. Vidroiu S. T. . (2025). Hematological inflammatory markers and chronic diseases: Current evidence and future perspectives. Hemato 6, 42. doi: 10.3390/HEMATO604004230654563

[B16] Dumitrescu F. Keiko Gunawan C. Sumarpo A. Rengga Indrati A. (2026). Immune dysregulation in HIV-TB co-infection: Role of cytokines and T cell biomarkers—a narrative review. Pathogens 15, 51. doi: 10.3390/PATHOGENS15010051 41599035 PMC12845183

[B17] Ellaurie M. Rubinstein A. (1995). Elevated tumor necrosis factor-alpha in association with severe anemia in human immunodeficiency virus infection and Mycobacterium avium intracellulare infection. Pediatr. Hematol. Oncol. 12, 221–230. doi: 10.3109/08880019509029563 7640175

[B18] Fang G. Mo Y. Bao Y. Gao C. Pan Q. Lu T. (2025). The clinical value of PLR, NLR, and MLR in predicting mortality risk in hospitalized patients. Front. Pharmacol. 16, 1736947. doi: 10.3389/fphar.2025.1736947 PMC12708535 41415568

[B19] Fuchs D. Zangerle R. Artner-Dworzak E. Weiss G. Fritsch P. Tilz G. P. . (1993). Association between immune activation, changes of iron metabolism and anaemia in patients with HIV infection. Eur. J. Haematol. 50, 90–94. doi: 10.1111/J.1600-0609.1993.TB00147.X 8440363

[B20] Gallagher P. G. (2022). Anemia in the pediatric patient. Blood 140, 571. doi: 10.1182/BLOOD.2020006479 35213686 PMC9373018

[B21] Gatechompol S. Kerr S. J. Cardoso S. W. Samaneka W. Tripathy S. Godbole S. . (2024). Monocyte to lymphocyte ratio and hemoglobin level to predict tuberculosis after antiretroviral therapy initiation. AIDS 38, 31–38. doi: 10.1097/QAD.0000000000003713 37696248 PMC10811616

[B22] Gelaw Y. Getaneh Z. Melku M. (2021). Anemia as a risk factor for tuberculosis: A systematic review and meta-analysis. Environ. Health Prev. Med. 26, 13. doi: 10.1186/S12199-020-00931-Z 33485299 PMC7824931

[B23] Gibellini D. Clò A. Morini S. Miserocchi A. Ponti C. Re M. C. (2013). Effects of human immunodeficiency virus on the erythrocyte and megakaryocyte lineages. World J. Virol. 2, 91–101. doi: 10.5501/wjv.v2.i2.91 24175233 PMC3785048

[B24] Han L. Bi L. Li X. (2025). The diagnostic value of decreased levels of inflammatory markers for the state of hepatitis C virus (HCV) infection. PloS One 20. doi: 10.1371/JOURNAL.PONE.0332105 40934209 PMC12425221

[B25] He Q. He Q. Qin X. Li S. Li T. Xie L. . (2016). The relationship between inflammatory marker levels and hepatitis C virus severity. Gastroenterol. Res. Pract. 2016, 2978479. doi: 10.1155/2016/2978479 28090206 PMC5206414

[B26] Isanaka S. Mugusi F. Urassa W. Willett W. C. Bosch R. J. Villamor E. . (2012). Iron deficiency and anemia predict mortality in patients with tuberculosis3. J. Nutr. 142, 350–357. doi: 10.3945/JN.111.144287 22190024 PMC3260062

[B27] Jin M. Wang Y. Li J. Wu Z. Liu X. Wang H. . (2023). Anemia is independently associated with mortality in people living with human immunodeficiency virus/acquired immune deficiency syndrome: A propensity score matching-based retrospective cohort study in China. Front. Med. (Lausanne) 10, 1055115. doi: 10.3389/fmed.2023.1055115 PMC9975156 36873867

[B28] Kamvuma K. Hamooya B. M. Munsaka S. Masenga S. K. Kirabo A. (2024). Mechanisms and cardiorenal complications of chronic anemia in people with HIV. Viruses 16, 542. doi: 10.3390/V16040542 38675885 PMC11053456

[B29] Kerkhoff A. D. Wood R. Cobelens F. G. Gupta-Wright A. Bekker L. G. Lawn S. D. (2015). The predictive value of current haemoglobin levels for incident tuberculosis and/or mortality during longterm antiretroviral therapy in South Africa: A cohort study. BMC Med. 13, 1–13. doi: 10.1186/s12916-015-0320-9 25889688 PMC4411796

[B30] Ketut Agus Somia I. Merati T. P. Made Bakta I. Manuaba I. B. P. Yasa W. P. S. Dewa Made Sukrama I. . (2019). High levels of serum IL-6 and serum hepcidin and low CD4 cell count were risk factors of anemia of chronic disease in HIV patients on the combination of antiretroviral therapy. HIV AIDS (Auckl) 11, 133. doi: 10.2147/HIV.S195483 31213927 PMC6549426

[B31] Koval D. Bielosludtseva K. Koval D. Bielosludtseva K. (2022). Neutrophil-to-lymphocyte ratio (NLR) as a marker of COVID-19-associated pneumonia progression. Eur. Respir. J. 60, 3533. doi: 10.1183/13993003.CONGRESS-2022.353340074277

[B32] Lipshultz H. M. Hileman C. O. Ahuja S. Funderburg N. T. McComsey G. A. (2015). Anemia is associated with monocyte activation in HIV-infected adults on antiretroviral therapy. Antivir Ther. 20, 521. doi: 10.3851/IMP2940 25668820 PMC4531107

[B33] Man Q. Liu S. Huang Y. Yang Y. Wen J. Fan L. . (2025). Association between CBC-derived inflammatory biomarkers and mortality in osteoarthritis patients: Results from NHANES. Medicine 104, e45024. doi: 10.1097/MD.0000000000045024 41189235 PMC12537135

[B34] Mata-Marín J. A. Gaytán-Martínez J. E. Martínez-Martínez R. E. Arroyo-Anduiza C. I. Fuentes-Allen J. L. Casarrubias-Ramirez M. (2010). Risk factors and correlates for anemia in HIV treatment-naïve infected patients: A cross-sectional analytical study. BMC Res. Notes 3, 230. doi: 10.1186/1756-0500-3-230 20727136 PMC2941498

[B35] Mayito J. Meya D. B. Rhein J. Sekaggya-Wiltshire C. (2020). Utility of the monocyte to lymphocyte ratio in diagnosing latent tuberculosis among HIV-infected individuals with a negative tuberculosis symptom screen. PloS One 15. doi: 10.1371/JOURNAL.PONE.0241786 33166312 PMC7652277

[B36] McNamara L. A. Collins K. L. (2011). Hematopoietic stem/precursor cells as HIV reservoirs. Curr. Opin. HIV AIDS 6, 43–48. doi: 10.1097/COH.0B013E32834086B3 21242893 PMC3045752

[B37] Morini S. Musumeci G. Bon I. Miserocchi A. Alviano F. Longo S. . (2016). HIV-1 gp120 impairs the differentiation and survival of cord blood CD34+ HPCs induced to the erythroid lineage summary. New Microbiologica 39, 13–23. 26922982

[B38] Oh E. S. You Z. Nowak K. L. Jovanovich A. J. (2022). Association of monocyte count and monocyte/lymphocyte ratio with the risk of cardiovascular outcomes in patients with CKD. Kidney360 3, 657–665. doi: 10.34067/KID.0007922021 35721602 PMC9136887

[B39] Oramasionwu C. U. Koeller J. M. Lawson K. A. Brown C. M. Morse G. D. Frei C. R. (2012). The state of disparities in opportunistic infection prophylaxis for blacks with HIV/AIDS. Med. Care 50, 920–927. doi: 10.1097/MLR.0B013E31826C85D1 23047780 PMC3470654

[B40] Pitchumoni C. S. Brun A. (2025). “ HIV and AIDS,” in Geriatric Gastroenterology, (New York, NY: Springer New York) 659–666. doi: 10.1007/978-1-4419-1623-5_71

[B41] Qiu C. Liu S. Li X. Li W. Hu G. Liu F. (2023). Prognostic value of monocyte-to-lymphocyte ratio for 90-day all-cause mortality in type 2 diabetes mellitus patients with chronic kidney disease. Sci. Rep. 13, 1–8. doi: 10.1038/s41598-023-40429-6 37573470 PMC10423199

[B42] Renelt S. Schult-Dietrich P. Baldauf H. M. Stein S. Kann G. Bickel M. . (2022). HIV-1 infection of long-lived hematopoietic precursors *in vitro* and *in vivo*. Cells 11. doi: 10.3390/CELLS11192968 36230931 PMC9562211

[B43] Sullivan P. S. Hanson D. L. Chu S. Y. Jones J. L. Ward J. W. (1998). Epidemiology of anemia in human immunodeficiency virus (HIV)-infected persons: Results from the Multistate Adult and Adolescent Spectrum of HIV Disease Surveillance Project. Blood 91, 301–308. doi: 10.1182/BLOOD.V91.1.301 9414298

[B44] Teer E. Mukonowenzou N. C. Essop M. F. (2025). HIV, inflammation, and immunometabolism: A model of the inflammatory theory of disease. Viruses 17, 839. doi: 10.3390/V17060839 40573430 PMC12197572

[B45] Tesfaye S. Hirigo M. Jember D. Shifeta M. Ketema W. (2023). Burden of anemia among human immunodeficiency virus-positive adults on highly active antiretroviral therapy at Hawassa University Compressive Specialized Hospital, Hawassa, Ethiopia. Anemia 2023. doi: 10.1155/2023/2170447 37868338 PMC10590265

[B46] Unaids (2024). Middle East and North Africa regional profile — 2024 global AIDS update The Urgency of Now: AIDS at a Crossroads Middle East and North Africa Regional Profile — 2024 Global AIDS Update the Urgency of Now: AIDS at a Crossroads. Available online at: http://hivfinancial.unaids.org/hivfinancialdashboards.html (Accessed May 13, 2025).

[B47] Vajpayee M. Mohan T. (2011). Current practices in laboratory monitoring of HIV infection. Indian J. Med. Res. 134, 801. doi: 10.4103/0971-5916.92627 22310815 PMC3284091

[B48] Veronese N. Segala F. V. Carruba L. La Carrubba A. Pollicino F. Di Franco G. . (2023). Anemia as a risk factor for disease progression in patients admitted for COVID-19: Data from a large, multicenter cohort study. Sci. Rep. 13, 1–7. doi: 10.1038/s41598-023-36208-y 37270578 PMC10239032

[B49] Wacka E. Nicikowski J. Jarmuzek P. Zembron-Lacny A. Wacka E. Nicikowski J. . (2024). Anemia and its connections to inflammation in older adults: A review. J. Clin. Med. 13, 2049. doi: 10.3390/JCM13072049 38610814 PMC11012269

[B50] Wang D. Hou X. Yu X. Wang T. Ye Z. Li J. . (2021). Plasma hemoglobin and the risk of death in HIV/AIDS patients treated with antiretroviral therapy. Aging (Albany NY) 13, 13061. doi: 10.18632/AGING.202987 33971620 PMC8148493

[B51] Weiss G. Ganz T. Goodnough L. T. (2019). Anemia of inflammation. Blood 133, 40–50. doi: 10.1182/BLOOD-2018-06-856500 30401705 PMC6536698

[B52] Yang J. Yan B. Yang L. Li H. Fan Y. Zhu F. . (2018). Macrocytic anemia is associated with the severity of liver impairment in patients with hepatitis B virus-related decompensated cirrhosis: A retrospective cross-sectional study. BMC Gastroenterol. 18. doi: 10.1186/S12876-018-0893-9 30384828 PMC6211489

[B53] Zauli G. Davis B. Re M. Visani G. Furlini G. La Placa M. (1992). tat protein stimulates production of transforming growth factor-β1 by marrow macrophages: A potential mechanism for human immunodeficiency virus-1–induced hematopoietic suppression. Blood 80, 3036–3043. doi: 10.1182/BLOOD.V80.12.3036.3036 1281686

[B54] Zhang X. Rovin B. H. (2010). Hepcidin expression by human monocytes in response to adhesion and pro-inflammatory cytokines. Biochim. Biophys. Acta 1800, 1262. doi: 10.1016/J.BBAGEN.2010.08.005 20801192 PMC2967603

